# Supplementary material for: Patterns of ASFV Transmission in Domestic Pigs in Serbia
Source: Pathogens. 2023 Jan 16;12(1):149. doi: 10.3390/pathogens12010149 (PMC9862985; doi:10.3390/pathogens12010149)
Supplement: Supplementary file 1 [file pathogens-12-00149-s001.zip › supplementary1. List of questions.pdf]

SURVEY QUESTIONNAIRE

I Farm information:

1. What kind of farm do you have?
   a) Backyard farm
   b) Commercial farm
2. Do you practice mixed farming (different animal species on the same farm)
   a) Y (If the answer is yes, which animal species)
   b) N
3. How many pigs do you house? (Number required)
4. What categories of pigs do you house?
   A) Fattening pigs
   B) Sows
   C) Boars
   D) Piglets
5. How many workers do you have?
6. Do you employ workers or do your family members work on the farm?
   a) Outside workers
   b) Family members
   c) Both
7. Are there other backyard farms or commercial farms near yours?
   a) Y
   b) N
8. Do you have biosecurity measures in place?
   a) Y (If yes what do they include)
   b) N

II Information regarding the feed used:

1. What kind of pig feed do you use?
   a) Commercial
   b) I make my own
   c) Both
2. How often do you feed your pigs?
   a) Once a day
   b) Twice a day
   c) Ad libitum
3. Have you recently acquired new pig feed?
   a) Y
   b) N
4. Do you practice swill feeding?
   a) Y
   b) N
5. Do you practice pasture farming during the summer?
   a) Y
   b) N

III Information regarding hunting, and veterinary visits:

1. Are you a hunter?
   a) Y
   b) N

2. Have you had recent contact with hunters?
   a) Y
   b) N
3. Did you acquire any meat from wild boars?
   a) Y
   b) N
4. Do you have good contact with your local veterinary service?
   a) Y
   b) N

IV African swine fever awareness:

1. Have you heard of African swine fever?
   a) Y
   b) N
2. Did you know that African swine fever has been confirmed in your municipality?
   a) Y
   b) N
3. Can you name some clinical signs that you should watch for?
   a) Y – which ones
   b) N
4. What should you do when you notice that your animal is sick?
   a) Call the local veterinary service immediately
   b) Treat the animal myself with antibiotics
   c) Wait a couple of days, and if the animal remains sick treat it yourself
   d) Wait a couple of days, and if the animal remains sick call the veterinary service
   e) Call the local veterinary service if the animal dies, or others become ill
   f) Do nothing
5. What do you think your role is in preventing ASF spread?
6. Did you know that the government compensates all farm holders whose swine are afflicted with African swine fever?
   a) Y
   b) N
