# Supplementary material for: Patterns of ASFV Transmission in Domestic Pigs in Serbia
Source: Pathogens. 2023 Jan 16;12(1):149. doi: 10.3390/pathogens12010149 (PMC9862985; doi:10.3390/pathogens12010149)
Supplement: Supplementary file 1 [file pathogens-12-00149-s001.zip › Supplementary2. Answers of the questionaree.pdf]

I Farm information		
Question	Answer	Percentage
What kind of farm do you have?	Backyard farm	100 %
	Commercial farm	0
Do you practice mixed farming?	Yes	83% (Mostly ruminants, cows, sheep, and goats)
	No	17%
How many pigs do you house?	1-20	Average number: 15
What categories of pigs do you house	Fattening pigs	93%
	Sows	65%
	Boars	1%
	Piglets	76%
How many workers do you have?	1--5	Average number: 2.5
Do you employ workers or do your family members work on the farm?	Outside workers	12%
	Family members	88%
	Both	12%
Are there other backyard farms or commercial farms near yours?	Yes	95%
	No	5%
*Do you have biosecurity measures in place?	Yes	33%
	No	67%

*Answers diverging from those offered by the questionnaire.

Most farm owners were not informed on what constitutes correct biosecurity measures, thus this part of the questionnaire should be considered in a broader concept.

II Information regarding the feed used		
Question	Answer	Percentage
What kind of pig feed do you use?	Commercial	68%
	I make my own	71%
	Both	83%

How often do you feed your pigs	Once a day	13%
	Twice a day	73%
	Ad libitum	14%
Have you recently acquired new pig feed?	Yes	41%
	No	59%
Do you practice swill feeding?	Yes	8%
	No	92%
Do you practice pasture farming during the summer?	Yes	55%
	No	45%

**III Information regarding hunting, and veterinary visits**		
Question	Answer	Percentage
Are you a hunter?	Yes	0%
	No	100%
Have you had recent contact with hunters?	Yes	37%
	No	63%
Did you acquire any meat from wild boars?	Yes	8%
	No	92%
Do you have good contact with your local veterinary service?	Yes	74%
	No	36%

**IV African swine fever awareness**		
Question	Answer	Percentage
Have you heard of African swine fever?	Yes	32%
	No	78%
*Did you know that African swine fever has been confirmed in your municipality? (From which source)	Yes	86%
	No	14%
*Can you name some clinical signs that you should watch for?	Yes	Comments are under the table
	No	

What should you do when you notice that your animal is sick?	Call the local veterinary service immediately	23%
	Treat the animal myself with antibiotics	42%
	Wait a couple of days, and if the animal remains sick call the veterinary service	29%
	Call the local veterinary service if the animal dies, or another becomes ill	4%
	Do nothing	0%
*What do you think your role is in preventing the ASF spread?		Comments are under the table
*Did you know that the government compensates all farm holders whose swine are afflicted with African swine fever?	Yes	9%
	No	91%

*Answers diverging from those offered by the questionnaire.

Did you know that African swine fever has been confirmed in your municipality? (From which source): Most farmers were informed of positive cases of ASF in their municipality, but when asked about the source, most stated "word of mouth", and that no government authority made the information public.

Can you name some clinical signs that you should watch for?:

Answers: Since the answers were not restricted by the questioner, most were guesses at correct answers. In some cases, farmers stated that they were not informed on what to look for.

What do you think your role is in preventing the ASF spread?:

Answers: The majority of farm owners consider that they do not have a responsibility towards stopping the spread of ASF and that it's a government issue.

Did you know that the government compensates all farm holders whose swine are afflicted with African swine fever?:

Answers: The majority of farm owners did not know that the government has an established program for refunding those farm owners who conducted all necessary biosecurity measures or did not believe that the government refunded the owners.
