# Supplementary material for: Patterns of ASFV Transmission in Domestic Pigs in Serbia
Source: Pathogens. 2023 Jan 16;12(1):149. doi: 10.3390/pathogens12010149 (PMC9862985; doi:10.3390/pathogens12010149)
Supplement: Supplementary file 1 [file pathogens-12-00149-s001.zip › Table S3. Sequences used in the B646L gene phylogenetic study.pdf]

Table S1. The accession numbers of the B646L gene from the NCBI were used for the alignment with sequences from this study.

Accession number	Country	Year	Domestic pig/Wild boar
KJ195685	Russia	2012	Domestic pig
AF301537	Portugal	1957	Domestic pig
MW451091	Vietnam	2019	Domestic pig
MW451093	Vietnam	2020	Domestic pig
MZ682070	Vietnam	2020	Domestic pig
OP019317	Russia	2021	Domestic pig
OM986194	China	2021	Domestic pig
MW889890	Czech Republic	2018	Wild boar
LR536725	Belgium	2018	Wild boar
LR722599	Moldova	2017	Wild boar
LR722600	Czech Republic	2017	LR722600
LR899193	Germany	2020	Domestic pig
MN715134	Hungary	2018	Wild boar
MT847621	Poland	2017	Wild boar
MT847622	Poland	2017	Wild boar
MT851941	Indonesia	2020	Domestic pig
MT840356	Russia	2019	Domestic pig
MK189456	China	2018	Wild boar
KJ496127	Ukraine	2014	Wild boar
OQ060619	Serbia	2021	Domestic pig
OQ060620	Serbia	2021	Domestic pig

OQ060621	Serbia	2021	Wild boar
OQ060622	Serbia	2021	Domestic pig
OQ060623	Serbia	2021	Domestic pig
OQ060624	Serbia	2021	Domestic pig
OQ060625	Serbia	2021	Domestic pig
OQ060626	Serbia	2021	Wild boar
OQ060627	Serbia	2021	Domestic pig
OQ060628	Serbia	2021	Domestic pig
OQ060629	Serbia	2021	Domestic pig
OQ060630	Serbia	2021	Wild boar
OQ060631	Serbia	2021	Wild boar
OQ060632	Serbia	2021	Wild boar
OQ060633	Serbia	2021	Wild boar
OQ060634	Serbia	2021	Wild boar
JX857510	Russia	2007	Wild boar
JX857511	Russia	2008	Wild boar
JX857512	Russia	2008	Domestic pig
JX857518	Russia	2009	Domestic pig
JX857519	Russia	2009	Domestic pig
JX857521	Ukraine	2012	Domestic pig
MT771051	South Korea	2019	Wild boar
OM986132	China	2019	Domestic pig
