# Supplementary material for: Patterns of ASFV Transmission in Domestic Pigs in Serbia
Source: Pathogens. 2023 Jan 16;12(1):149. doi: 10.3390/pathogens12010149 (PMC9862985; doi:10.3390/pathogens12010149)
Supplement: Supplementary file 1 [file pathogens-12-00149-s001.zip › Table S4. Sequences used in the B602L phylogenetic study.pdf]

Table S2. The accession numbers of the B602L gene from the NCBI were used for the alignment with sequences from this study.

Accession number	Country	Year	Domestic pig/Wild boar
OQ060635	Serbia	2021	Domestic pig
OQ060636	Serbia	2021	Domestic pig
OQ060637	Serbia	2021	Wild boar
OQ060638	Serbia	2021	Domestic pig
OQ060639	Serbia	2021	Domestic pig
OQ060640	Serbia	2021	Domestic pig
OQ060641	Serbia	2021	Domestic pig
OQ060642	Serbia	2021	Wild boar
OQ060643	Serbia	2021	Domestic pig
OQ060644	Serbia	2021	Domestic pig
OQ060645	Serbia	2021	Domestic pig
OQ060646	Serbia	2021	Wild boar
OQ060647	Serbia	2021	Wild boar
OQ060648	Serbia	2021	Wild boar
OQ060649	Serbia	2021	Wild boar
OQ060650	Serbia	2021	Wild boar
OQ060635	Serbia	2021	Domestic pig
FR682468	Georgia	2007	Domestic pig
KY372397	Russia	2015	Domestic pig
KY372398	Russia	2016	Domestic pig
LR722599	Moldova	2017	Wild boar

LR899193	Germany	2020	Wild boar
LS478113	Estonia	2014	Wild boar
MK628478	Lithuania	2014	Wild boar
MN194591	Ukraine	2016	Domestic pig
MN715134	Hungary	2018	Wild boar
MN809122	Romania	2017	Domestic pig
MT847620	Poland	2017	Wild boar
MT847622	Poland	2017	Wild boar
MW361944	China	2019	Domestic pig
MW451102	Vietnam	2019	Domestic pig
MW451105	Vietnam	2020	Domestic pig
OM966720	Russia	2018	Wild boar
ON075797	South Korea	2019	Wild boar
ON108571	Italy	2022	Wild boar
OP628183	South Korea	2020	Wild boar
